# Proactive Personality and Innovative Work Behavior: Through the Juxtapose of Schumpeter's Theory of Innovation and Broaden-And-Build Theory

**DOI:** 10.3389/fpsyg.2022.927458

**Published:** 2022-06-13

**Authors:** Weizheng Li, Shabeeb Ahmad Gill, Yang Wang, Muhammad Adeel Safdar, Muhammad Ramzan Sheikh

**Affiliations:** ^1^School of Economics and Management, Weifang University, Weifang, China; ^2^Department of Personnel and Administration, NFC Institute of Engineering and Fertilizer Research Faisalabad, Faisalabad, Pakistan; ^3^School of Economics, Bahauddin Zakariya University, Multan, Pakistan

**Keywords:** proactive personality, open innovation, perceived insider status, innovative work behavior, theory of innovation, broaden-and-build theory, information technology

## Abstract

This study aimed to unfold the implicit interplay of open innovation (OI) and perceived insider status (PIS) between the relationship of proactive personality (PP) and innovative work behavior (IWB). The phenomenon studied the moderated mediation of OI and PIS through the combined optic of the theory of innovation and the broaden-and-build theory. The nature of this study was post-positivist. The two-step approach of structural equation modeling was implemented. First, quantitative data were collected through an online questionnaire from the employees of IT industries in China. The study sample consisted of 460 responses used for data analysis in SPSS and AMOS version 26. This study was based on mediated moderation, which was statistically similar to Model 15 of the process macro. There were six hypotheses based on the theoretical framework. The result of H6 was rejected, which demonstrated that the conditional direct effect of OI and PIS mediated moderation on PP and IWB. The results comprehensively testified to the theoretical framework.

## Introduction

In the current industrial generation, innovation is the most dynamic component in producing highly competitive and innovative products and services to meet the requirements of modern customers (Tran et al., 2020). The fundamental reason for fame is that innovation has become the frontline component in organizational research and practice. Among multiple business process resources, employees' innovative work behavior (IWB) has emerged as a substantive factor (Kumar et al., 2022). Although, in the current study, antiquities have established the positively significant relationship of IWB with leadership (Kim and Koo, 2017), work climate (Baradarani and Kilic, 2018), OI (Luhgiatno and Dwiatmadja, 2020), PIS (Zhang et al., 2021), and proactive personality (PP) (Ahmad et al., 2021b; Zhang et al., 2021). PP is essential for individual and organizational success. While several investigations have shown that an Employee PPcan determine work results (Hogan and Holland, 2003; Alikaj et al., 2021), a smaller stream of work has looked into the impact of different personality traits on Employee IWB. For example, Zhou and George (2001) found a favorable relationship between PP and IWB. Researchers have also looked at the relationship between PP and IWB. A person with a PP, according to Bateman and Crant (1993) is “largely unfettered by specific circumstances and who impacts environmental change” (p. 105). This PP measures how likely employees are to seek out and take advantage of possibilities for transformation (Li et al., 2017a).

Individuals with a PP are motivated to seek more comprehensive and innovative solutions to complex procedures or methods that they may consider ineffective to enhance their existing conditions rather than passively adapting to all parts of the organizational ecological system (Parker et al., 2010). Employees who use IWB at work develop ideas that might be applied to an organization's products or services. As a result, businesses can use their employees' innovative ideas to respond to market shifts or seize market opportunities, boosting their competitive advantage (Shalley and Gilson, 2004). Employee IWB has been discovered to play an essential role in influencing organizational effectiveness (Alikaj et al., 2021). As a result, we believe it is similarly vital to investigate IWB from the variety of knowledge and workplace circumstances that businesses provide for their workforce. Employees' ability to produce and execute innovative solutions, standardize processes, and increase teamwork is closely tied to their IWB (Onne, 2000). Employees are an essential element of a business's innovation process; thus, enabling their IWB is critical. Consequently, IWB is critical to business sustainability (Jankelová et al., 2021).

Businesses now use open innovation (OI) for internal innovative management operations. It also examines how initiatives affect information technology (IT) firms (Moradi et al., 2021). Due to precursors, a new approach to an innovation known as “OI” has emerged. OI adds value to businesses by encouraging knowledge exchange within and between organizations. OI is founded on the assumption that new ideas are not confined to a few firms or units (Chesbrough and Garman, 2009) but are dispersed across the economy. OI is defined as the systematic investigation, preservation, and dissemination of knowledge inside and outside organizational boundaries in keeping with the tendency to develop new business alliances in intra- and extra-organizational collaborations (Cronin, 2020). The bulk of research by Chesbrough et al. (forthcoming) was undertaken at the level of organizations and organizational networks, with the primary goal of understanding the antecedents, processes, and potential benefits and drawbacks of OI (Bogers et al., 2018). Organizations can gain from implementing an OI strategy in terms of revenue, capacity to deal with emerging market issues (Gassmann et al., 2010; Bachem and Casey, 2018; Bigliardi et al., 2021), and the ability to recognize new product combinations according to scholarly data (Almirall and Casadesus-Masanell, 2010). While the literature provides a thorough overview of the possible benefits of OI, it only provides a limited understanding of how the OI process might be handled and led (Edelbroek et al., 2019).

Kuo et al. (2015) believed that employees, managers, and organizations are the primary sources of their impressions of the atmosphere, in which workplace they belong. Consequently, we investigate how employees' general attitudes toward the company, such as perceived insider status (PIS), affect their IWB because of satisfaction at the individual level. Take into consideration the following objectives together to better grasp how IWB differs depending on the characteristics of the PIS objects (Brady et al., 2017). Furthermore, considering PIS as a boundary condition for the IWB links, we discover the importance of individual differences in outlining why employees participate in workplace PIS. Therefore, our fiction highlights the selfish motivations of employees affected by how organizations and managers treat them, PIS patterns based on items, and employees' unique characteristics at IWB.

Yin et al. (2021) only introduce PIS to explain the mechanism of PP and employee innovative behavior. In future studies, more mechanisms from different viewpoints should be investigated. They did not unfold the moderating role of PIS in the outcome variable; in this study, the scholar measured the moderating effect of PIS on the indirect mediated relationship. For example, OI and assets encouraging IWB could function in a feedback system, encouraging WIB. Although the earlier research concentrated on the mechanics driving the link between PP and IWB rather than how various facets of PP are related to IWB, they noted that it would be helpful to investigate the differences in the effects of these three aspects on employee IWB. Similarly, antecedents vacant a drift in knowledge to investigate PP on IWB through the mediation of OI (Qureshi and Ahmed, 2021).

### Study Gap

In the previous research studies, IWB has been investigated with many intervening variables: trust in leadership (Hoang et al., 2022) and psychological safety (Ahmad et al., 2021b). Although we found that the affective states and creative self-efficacy (Li et al., 2017a), error management climate and self-efficacy (Pan et al., 2021) have been investigated as the mediators between the relationship between PP and IWB. However, Dionne et al. (2002) proposed studying openness to experience and general mental ability as mediators in the future. The current study found no evidence from literature about the mediating role of OI between PP and IWB. To fill this colossal literature gap, we investigated the mediating role of OI between PP and IWB in the current settings.

PIS has been studied as a positive relationship as the outcome variable with PP (Dionne et al., 2002), as a mediator with organizational citizenship behavior and found no mediation (Caron et al., 2019), as the mediator between living a calling and psychological well-being and resulted in a partial mediation (Kang et al., 2021). On the other hand, PIS has been studied as an independent variable on IWB and established a significantly positive relationship (Wang et al., 2017). The dependent relationship of PIS with PP and the independent relationship of PIS on IWB has been established in published scientific work. So, we propel to study the mediated moderation of OI and PIS on the indirect and direct relationship of PP and IWB.

The PP of employees could have an explicitly positive impact on their IWB to meet the current market needs; it could enhance the economic growth, and, ultimately, the organization will become prosperous. The employees of the IT industry in China could enhance the business growth by triggering the IWB through the PP. In many studies, Truxillo et al. (2012); Ruiz Moreno et al. (2021); and Zhang et al. (2021) have conceptualized and accepted that PP positively impacts employee outcomes but ignored the IWB, so this study aimed to fill a vital literature gap. Unfortunately, the employees in the IT sector are missing PP's features, which is the fundamental reason they are unable to meet the standards of innovative products; resultantly, our competitors are cashing the scenario, from here the Schumpeter's theory of innovation luminous the path.

In the current era of high-tech development, especially in the IT sector, the rivulet of competition is flowing with a proclivity. A stride propelled into IT without the propensity for innovation is nearly impossible based on Schumpeter's theory of innovation (Sweezy, 1943; Swedberg, 2015). Employees' IWB is grounded in the Broden-and-build theory because positive emotions provide the base to surge innovation in the workplace (Fredrickson, 1998). This study brought the juxtaposition of innovation theory and Broden-and-build theory to frame this organizational phenomenon.

## Review of Literature and Hypotheses Development

### Theory and Conceptual Linkage

An economic revolution was brought about through entrepreneurial function, which entails recognizing and bringing out new opportunities in the financial world, according to the theory of entrepreneurship and innovation presented in 1926 (Mathews, 2002). Nevertheless, according to Professor Schumpeter, the entrepreneur is now “the middleman” between producers and customers. Because the entrepreneurial purpose now acts through social contact, Schumpeter would primarily think of it as an example of interdependence rather than causation (as he previously did in 1911). This point of view is articulated by both entrepreneurship and the theory of innovation, published in 1926. Additionally, Schumpeter would continually rely on his emergent notion of entrepreneurship as a case of social interaction that usually avoids conceptualization in terms of cause and effect across his publications from 1926 onwards. It is worth noting that Schumpeter's dichotomy between causality and social interaction arose from fundamental difficulties in conceiving social interaction in terms of causality, which persist in evolutionary explanations of economic reform (Sweezy, 1943; Swedberg, 2015).

However, there have been tales of the term “innovation,” indicating something uncommon since the late 1880s; none of the earliest innovators have been as crucial as Schumpeter. According to his innovation theory, purchase intentions are predetermined and do not change independently. As a result, they are unable to affect economic reform. Furthermore, customers passively impact the economic development process (Sledzik, 2016). Throughout his economic development theory and subsequent research, Schumpeter defined development as a historical series of incremental changes, fueled mainly by innovation, which he classified into five categories. The first is the launch of a new product or a new species of an existing product; the second is the application of modern production methods or selling of a product or service (not yet proven in the industry). The third is to introduce a new market (a market for which a branch of the industry was not yet represented). The fourth is the acquisition of new raw material sources or semi-finished goods, and the fifth is the invention or destruction of a monopoly position (Bailey et al., 2018; Dalton and Logan, 2020).

It is momentous that the theory of innovation and Broden-and-build theory assimilate the factor of “innovation” and elucidate the coherent theoretical framework. The innovation theory emphasized the adoption and application of the innovative process of product manufacturing and service furnishing to extend the concept further. Furthermore, the Broden-and-build theory focused on the behavioral tendencies of the employees toward the innovation at work (Mishra et al., 2019). It also elaborates that the positive emotions of the employee broaden his/her cognitive actions, thus enhancing that employee's search for innovative ways of working (Slåtten, 2011). It has been explained as “positive emotions can broaden people's modes of thinking and, in doing so, make organizational members more creative” (Fredrickson, 1998, p. 174). Consequently, we propel this interesting and coherent theoretical framework for relationship development between PP and IWB through OI and PIS, albeit we claim that this is a unique theoretical model, which has never been tested amalgamating the theory of innovation and Broden-and-build theory.

### Proactive Personality and Innovative Work Behavior

Chien et al. (2021) PP refers to an individual's inclination to “scan for opportunities, show initiative, take action, and persevere until they reach closure by bringing about change” (Bateman and Crant, 1993, p. 104). PP describes someone “largely unfettered by situational circumstances and affects environmental changes” (Bateman and Crant, 1993, p. 105). In other words, a proactive individual needs to gather information, recognize and act on opportunities, and reshape the present situation or relocate to an ideal setting to effect substantial change in their workplace. Non-proactive persons, on the other hand, tend to wait for possibilities rather than actively shaping their settings silently. The notion of PP is predicated on the belief that some people are always trying to change their settings, change the world, and exercise direct jurisdiction over objective conditions. In contrast, others tend to conform to the status quo, adjust to the changes, and exercise secondary control over the prevailing situation (Weisz et al., 1990).

Onne (2000) elaborated IWB that “the intentional creation, introduction, and application of new ideas within a work role, group or organization, to benefit role performance, the group, or the organization (p. 288)”. Later on, Yuan and Woodman (2010) define it as an “employee's intentional introduction or application of new ideas, products, processes, and procedures” (p. 324). To generate alternative methods and ways by thinking out of the box is the way of IWB (Wojtczuk-Turek and Turek, 2015). Concerning the service sector, IWB is defined by Falih Bannay et al. (2020) as the new techniques and ways adopted by the front-line employees during customer dealing the conversion of potential problems into facilitation. According to the substitute for IWB, some contextual elements (individual, task, and organizational) may substitute or negate the impacts of output (Kerr and Jermier, 1978).

Previous research has established PP as a particular personality trait. In that, it stresses welcoming unique experiences (extraversion), goal completion (conscientiousness), and an exploration of the unfamiliar (openness), PP looks to be connected to some of the Big 5 personality traits. However, it is conceptually different: The essence of PP is the desire to exert control over one's surroundings by devising innovative solutions, assuming leadership roles in social networks, and accepting unpleasant sensations (Truxillo et al., 2012). Similar patterns may be seen at work: Specific individuals are continuously tracking difficulties, launching new projects, and developing concrete solutions, whereas others are reasonably content with the current situation and “go with the flow” (Bateman and Crant, 1993). The connection between PP and IWB has traditionally been predicated on personality traits. Such people are change-oriented, which means they like to alter the situation around them to fit their requirements better rather than merely adjusting or adapting to it. They enhance their performance by seeking new and more efficient ways of doing things (Choi and Thompson, 2005). That is consistent with the broaden-and-build theory; due to this search process, proactive individuals are more likely to demonstrate their IWB. Thus, based on the above discussion, we have formulated the following hypothesis:

*H1: PP is positively related to IWB*.

### Proactive Personality and Open Innovative

Yang et al. (2021) addressed that innovation is an essential aspect of an organization's survival in the related sector, but it is also the primary driver of long-term business growth. In essence, innovation is characterized as a process of repeatedly mixing a set of distinct abilities. Practical organizational innovation is the key to gaining and maintaining a competitive edge in changing and evolving environments (Sutanto, 2017). According to the concept of OI, companies and businesses should be more receptive and responsive to innovative processes. It has the potential to recruit more talent and transmit new ideas, research, and innovative technologies to other organizations (Sivakami, 2018). It enables external organizations to use potential internal ideas efficiently. OI implies that excellent ideas can come inside and outside the firm and be monetized inside or outside. Organizations offer interests and hazards by removing the barriers that separate organizational knowledge from the rest of the world. Including OI in a company's entrepreneurship process has numerous benefits. According to some research, embracing OI can boost product success rates by 50%, research efficiency by 60%, and internal development by 60% (Enkel et al., 2009).

A PP will employ their initiatives to modify their surroundings to construct a more desirable circumstance. According to a previous study (Bergeron et al., 2014), proactive employees are more likely to seek better solutions beyond their professional tasks to transform their existing situations. Substitutes for leadership, in other words, take the place of the PP influence by removing the necessity for leadership (Gok et al., 2017). Pursuing this line of reasoning, Gok et al. (2017) discovered that, while the PP is essentially redundant for someone with high moral awareness, it is helpful for those lacking moral recognition.

In this approach, the substitute refers to something or someone that reduces a leader's ability to influence subordinates' behaviors or attitudes toward OI (MacKenzie et al., 1993). Proactive employees are identifiable, conscientious, and persistent in bringing about change (Miscenko et al., 2017). Employees with a proactive attitude are minor subjects to social pressures and have more control over their work time. They take more aggressive actions and rely less on leadership cues to resolve work-related issues. Furthermore, proactive personnel come up with fresh ideas to improve work processes, demonstrate extraordinary dedication to goals, and put out high effort and performance, relying less on their superiors. The literature has already established a significant direct relation between PP and OI (Velez and Neves, 2018). Therefore, innovation with proactive personalities is much more likely to be creative than innovation with non-proactive personalities. The argument is coherent with one of the assumptions mentioned above of the theory of innovation; thus, the following hypothesis is established:

*H2. PP is positively related to OI*.

### Open Innovation and Innovative Work Behavior

Employees can be part of multiple interrelated phases of OI rather than a complete working line since the rebate offers to distinguish it. Although, at the last stage of implementation, a significant risk has to be taken by IWB before establishing an inclusive team to support the innovative idea (Xiong Chen and Aryee, 2007). Woefully, the body of established knowledge about IWB has different morphed and is still based on purported and needs to be elucidated (Bos-Nehles et al., 2017), even though it effectuates to surge and sustain (Tran et al., 2020) competitive advantage over competitors (Anderson et al., 2014; Hopwood, 2021; Mikhailova, 2021).

IWB was developed based on multiple interdependent elements like articulation, surge, marketing, and application of creating unique ideas (Onne, 2000) and employees' behavior to deal with and adopt these strategies (de Jong and den Hartog, 2010). A plethora of strategies could be adopted to do that, such as the uniformity of procedures, application of new technology and appropriate material, initiation of new tactics, enhancement of collaboration, and furnishing of the latest packages (Messmann and Mulder, 2012). The exploration, promotion, and actualization of unique ideas are the perceptible phases of IWB (Siregar et al., 2019).

Javed et al. (2019) and Nguyen et al. (2019) discussed that the antecedents of IWB individual, workgroup, and organization levels are job characteristics, work climate, individual differences, workgroup, personality, demands, values, and leadership directly related to IWB. Jankelová et al. (2021) emphasized that IWB never received the same consideration as the team and organization. Additionally, the research on OI and IWB has aspired to grow as the distance has shrunk due to globalization, economic variations, and competitive advantage (Bani-Melhem et al., 2018).

Employees play a critical role in the OI process. However, few studies look at the procedure from an employee's perspective (Bogers et al., 2018), leaving a gap in understanding how individual elements influence the organization's performance and how OI should always be organized and managed (Vanhaverbeke et al., 2014). Employee perceptions of the OI process are based on how well the operations of generating ideas, concept promotion, and idea realization are included in the teams in which they engage in the opportunity to proceed in OI (Rad et al., 2018). Through IWB, they integrate the OI capabilities in the manufacturing or furnishing process in the workplace. The phenomenon aligned the spirit of broad-and-build theory with the assumption of the theory of innovation. Hence, the following hypothesis is purported:

*H3: OI is positively related to IWB*.

### Proactive Personality, Open Innovation, Perceived Insider Status, and Innovative Work Behavior

We believe that PIS, which refers to a perceived relationship between employees and stakeholders in their organization, could be a criterion for the correlation between justice and pleasant IWB. Individuals' PIS relates to how they recognize themselves as insiders in their organization and is based on assessing their relative ranks inside the firm (Lapalme et al., 2009). Employees' perceptions of themselves as valuable members of their organization (i.e., insiders) can be linked to their distinct rewards and support (Armstrong-Stassen and Schlosser, 2011). Furthermore, speaking highly about benevolent action takers can help employees improve their reputations and meet their demand for status (Ellwardt et al., 2012). As a result, when employees perceive themselves as insiders due to their concern for the organization, they will plan to raise their own and others' positive work morale and esteem by distributing good news about their organization and supervisor. The percentage of employees who believe they are insiders rather than outsiders is the PIS (Stamper and Masterson, 2002). The principal stream of PIS, on the other hand, contends that the positive effects of PIS have outweighed the adverse effects. Employees who are “insiders”, long-term employees of the company, will develop a sense of obligation due to signals of inclusion or approval. Such sentiments of responsibility may inspire employees to contribute, freely communicate essential needs, and foster feelings of intimacy. Outsiders may not experience such emotions, at least not to the same degree. Therefore, PIS translates into a sense of perceived acceptance by their supervisor and other insiders, expressing the vital “belonging” emotion (McMillan and Chavis, 1986; Tan et al., 2021).

Jiang and Gu (2015) noted that proactive employees might experience a sense of accountability for change since they are given resources available by the business and are supposed to use those resources sustainably and make work-related advances to meet the organization's strategic goals. PP is a relative invention in disposition research, and it has gotten much attention in the management area since it was initially proposed. The person-environment relationship is viewed as a reciprocal process in this line of research, in which humans are not only sculptures generated by environmental factors, but also sculptors of their surroundings (Parker, 2016).

Providing a better working environment, increased job satisfaction, and better work-life are the aspiring consequences of the positive relationship between IWB and organization success (Lukes and Stephan, 2017). Usmanova et al. (2021) increased job satisfaction and communication efficiency, and harmonization of needs for jobs and resources of employees are the outcomes of IWB for individuals and organizations. In contrast, IWB leads to “individuals' behaviors directed toward initiating and intentionally introducing new and useful ideas, processes, products or procedures within a work role, group or organization” (de Jong and den Hartog, 2010). Onne (2000) elaborated that the IWB differs from creativity because it involves the invention, promotion, and implementation of innovative and beneficial ideas. Wu et al. (2014) IWB is critical to businesses since it helps with product design and favorably impacts organizational creativity, productivity, and longevity. Given its significance, previous research has investigated a variety of progenitors of IWB, such as job demands, job requirements, team and organizational climate, character, and leadership (Anderson et al., 2014), with leadership playing a significant and crucial role (Javed et al., 2019).

An excellent direct link between PP and IWB has been proved in previous studies, including a meta-analysis (Li et al., 2017b; Zhang et al., 2021). For example, a study by Kim et al. (2009) found that proactive employees in diverse Hong Kong organizations displayed higher levels of innovation. Furthermore, a study of Chinese high school teachers (Zhang et al., 2021) discovered that PP was linked to creative behavior. Therefore, the underlying mechanisms of the association between PP and creative behavior are investigated in this study (Fama and Jensen, 2019).

On either side, a PP should be included in the balanced combination since it can favorably impact OI involvement by pushing employees to be more creative, share information, and create independence (Jiang and Gu, 2015). They found that both facets of interplaying, OI, and PIS influence employee IWB, while PP employees may have a more significant impact. Rangus et al. (2017) stated that the term leadership had been acknowledged as OI's significant independent variable. This research investigates the influence of PIS with the intervening of the OI process by adding IWB.

On the one hand, PIS should be included in this mix since it has the potential to promote team cohesiveness through OI. On the other hand, PP can overcome employees' reluctance to participate in the OI process through flexible workplace systems to discover and use knowledge assets. Therefore, employees considered PIS are more likely to have high-quality interaction of broaden-and-built theory with the IWB employees from a theory of innovation standpoint through the intervening role of OI. In addition, employees with a high PIS level may be more influenced when they view their organization and supervisor as fair, owing to this high social external benchmark regarding innovative products on the direct and indirect relationships between PP and IWB (Wu et al., 2019). Thus, based on this scientific discussion, we have articulated the following mediated moderation hypotheses:

*H4: OI mediates the relationship between PP and IWB*.
*H5: PIS moderates the strength of the indirect relationship between PP and IWB through the mediation of OI. The relationship will be stronger for the higher PIS employees than for those lower in PIS*.
*H6: PIS moderates the strength of the direct relationship between PP and IWB through the mediation of OI. The relationship will be stronger for the higher PIS employees than for those lower in PIS*.

### Conceptual Model

**Figure 1:**
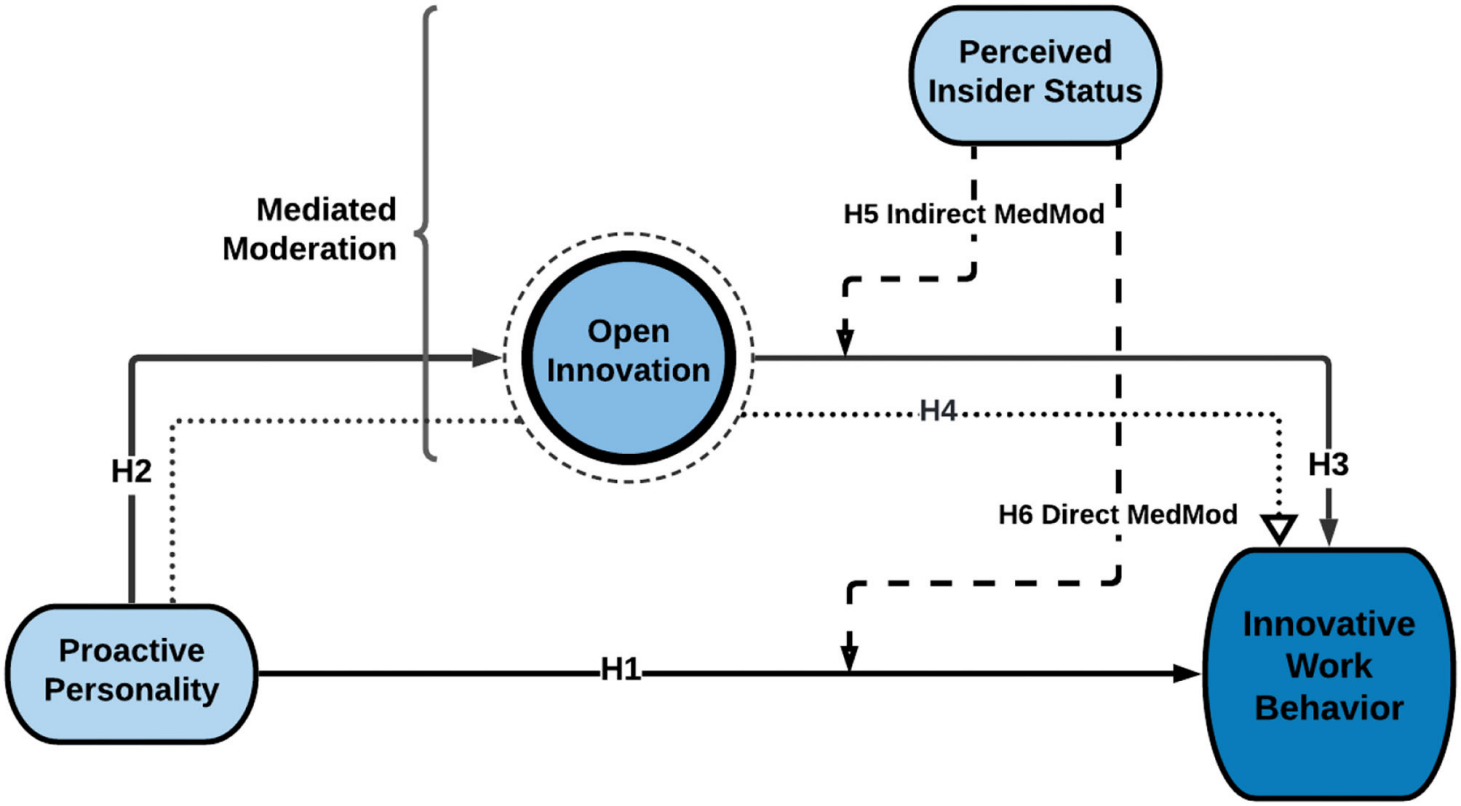
Conceptual model. Source: self-generated.

The conceptual model of the study is depicted in Figure 1.

## Materials and Methods

The plinth of this study permeated on post-positivist research philosophy. The phenomenon of this study was mind-dependent, and the only way to probe the truth was the scientific way of investigation. The empirical observations were used in the cross-sectional deductive settings (Krauss, 2015). Ontologically, the reality of this study was singular; epistemologically, it was objective. It has value-free axiology and quantitative survey methods used to conduct it (Bryman, 2016). Anderson and Gerbing (1988) elaborated a two-step approach to structural equation modeling (SEM) application. The application of SEM on parametric data through the general linear regression model (GLM) presented excellent strength of theory testing and development in business studies (Tabachnick et al., 2007). The data were analyzed through IBM SPSS AMOS version 26 (Byrne, 2016).

### Population and Sampling Technique

This study adopted the cluster sampling technique for data collection. It has implemented a multi-stage sampling approach; in the first stage, the population was divided into seven clusters; in the second stage, randomly three clusters have been selected out of seven (Shanghai, Nanjing, and Shandong) in China; in the third stage, by using simple random sampling (SRS) in each cluster, IT companies were designated. Finally, in the fourth stage, again, the employees were selected using SRS with the help of every company's human resource management office (Cochran, 1948; Heinisch, 1965; Naz et al., 2020).

### Sample Size and Procedure

The sample size was calculated through the sample size calculation software G^*^Power v 3.1. The parameter used was an effect size of 0.15 and power of 0.95, so the calculated sample size was 460 (Faul et al., 2007). An electronic format questionnaire has been floated through Google forms for data collection. To confirm the questionnaire validity after literature modification and to make it compatible with the target respondents, we pooled the opinions of more than 10 expert researchers. This study selected 25 IT companies based in (Shanghai, Nanjing, and Shandong), China. Finally, the electronic questionnaire in the English language was floated to 1,000 employees of 25 IT companies (Moradi et al., 2021); the employees' access was obtained from the human resources managers of the selected companies (Ahmad et al., 2021b). We received 538 responses from 1,000 respondents with a response rate of (53.8%). In Table 1, after scrutinizing all the responses, we discarded 50 responses due to incomplete information and finally selected 460 responses equivalent to our calculated sample size.

**Table 1:** Descriptive statistics.

	**Mean**	**Std. deviation**	**Variance**
Gender	1.15	0.355	0.126
Age	1.35	0.478	0.229
Experience	1.20	0.402	0.161

*Self-generated*.

### Measurement of Variables

This study purported five facets, one independent and one dependent, with the interplay of a single mediator and a moderator. Table 2 shows that five constructs were measured on a five-point Likert- scale (1–strongly disagree to 5–strongly agree). The only construct of “proactive personality” (PP) has been assessed with the 19-item scale (Bateman and Crant, 1993); later on, Seibert et al. (1999) validated its shorter version from 19 items to 10 items with a reliability of (17 items alpha = 0.88; 10 items alpha = 0.86). Recently, Mahmood et al. (2019) have revalidated the 10 items into 6 items with a reliability value of 0.78, which we used in this study for further investigation.

**Table 2:** Measures of the study.

	**Construct**	**Scale**	**Items**
Mediating variable	OI (OI)	Chesbrough and Garman, 2009; Lichtenthaler, 2009; Huang et al., 2013	10
Moderating	Perceived Inside Status (PIS)	Stamper and Masterson, 2002	10
Endogenous variable	IWB (IWB)	Scott and Bruce, 1994	09
Exogenous variable	PP(PP)	Bateman and Crant, 1993; Seibert et al., 1999; Mahmood et al., 2019	06

*Self-generated*.

The mediating construct of “OI” is based on the two concepts of literature: those are inbound with 4 items and outbound with 6 items; we used 10 items of OI together and dealt with it as a single construct in our study, and we measured OI through an adapted scale of Enkel et al. (2009), Lichtenthaler (2009), and Huang et al. (2013). To measure the moderating variable “PIS,” the study adopted the scale of Stamper and Masterson (2002) with slight modification; they developed 10 items of this construct with the initial reliability of alpha 0.88. “IWB” is the dependent variable; this study used the 9 items of the scale developed by Scott and Bruce (1994), and, consecutively, Onne (2000) applied and tested its reliability; which was proven to be highly reliable with alpha 0.95.

### Analytical Approach

To test the direct and indirect relationships between independent and outcome variables, a study tested 5 hypotheses based on mediated moderation. This study's theoretical model has statistical similarities with the process macro Model 15 (Barnidge and Zúñiga, 2017). That provided the statistical ground to test the single mediated moderation effects in a solo model. Indeed, testing Model 15 in a single shot is impossible in AMOS. However, we developed syntax and directed AMOS to analyze the critical paths commanded through user-defined estimates (Barnidge and Zúñiga, 2017).

The below formulated mathematical equations are commanded through a user-defined estimate in AMOS as syntax:

i) Direct effect without a mediator = *C*1;
ii) Indirect effect with a mediator= (A^*^B1);
iii) Direct effect without a mediator and a moderator = A^*^(*B*1+(*B*2^*^V);
iv) Indirect effect with a mediator and a moderator = *C*1+(*C*3^*^V).

**Figure 2:**
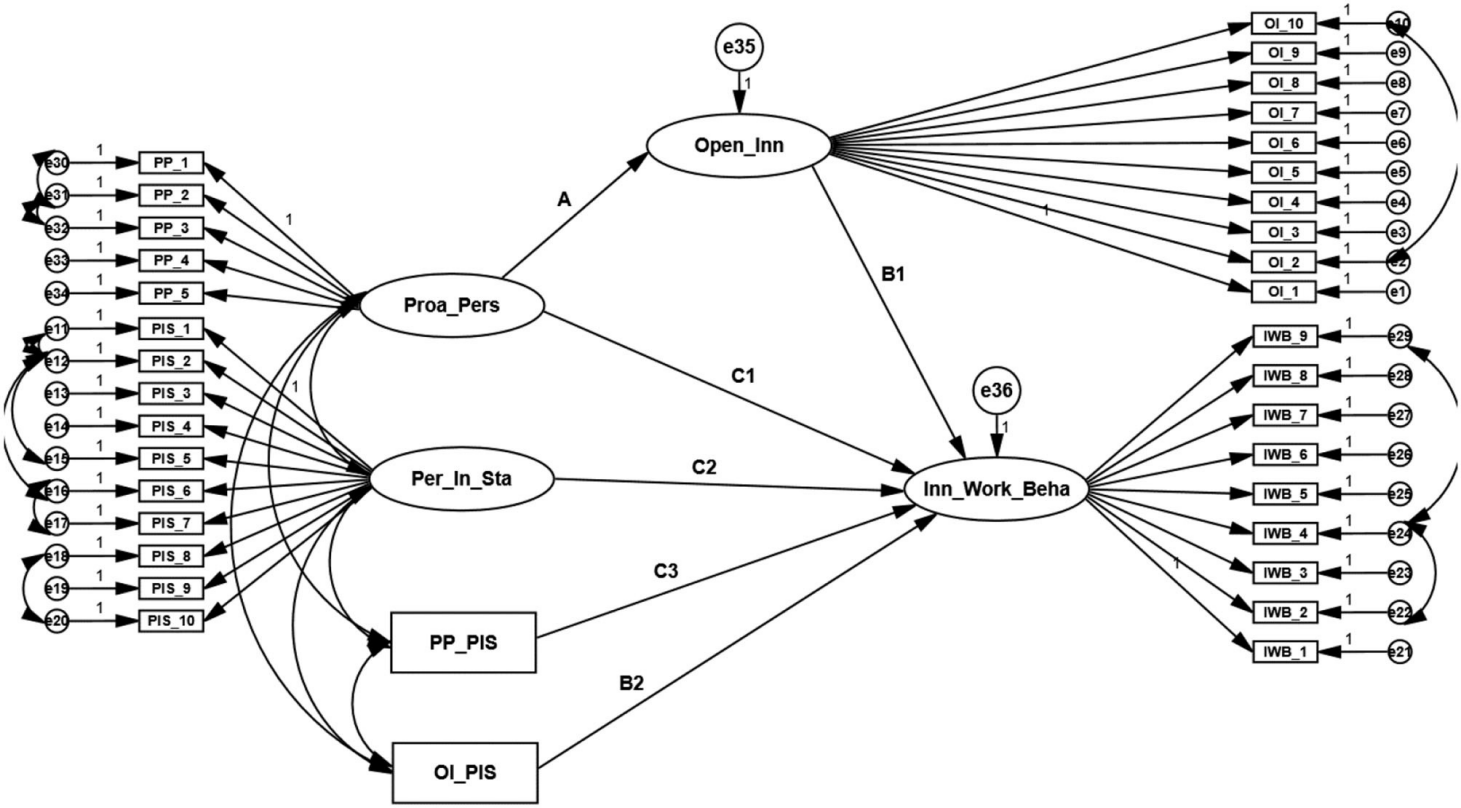
Structural model. Source: generated through AMOS.

*(Note: V* = *the standard deviation of a mediator)*; the path is illustrated in Figure 2. The direct and direct effects of PIS are estimated at high-, medium-, and low-strength levels.

## Results and Analysis

### Common Method Variance Bias

The study used to collect data of all the variables, including exogenous and endogenous, from the same respondents simultaneously; we avoided putting complex terminologies and asking vague statements. However, the chance of CMV could affect the results perniciously. So, to the inevitable influx of bias, the study used the single and latest approach, common latent factor (CLF) through AMOS, endorsed by Podsakoff et al. (2003), because the approach of Harman's single factor has been declared outdated by many studies due to its pernicious restrictions and limitations (Kumar and Shukla, 2019); and, recently, a study has recommended this aspersion (Gill et al., 2021). The difference between standardized regression weights after the inclusion and exclusion of CLF was difference *of* <0.2, so the data did not find evidence of CMV. The result of CVF was reported in Table 3. Furthermore, the study calculated variance inflation factors (VIF) to measure multicollinearity. VIF measured the correlation and its strength among independent variables; the highest value of VIF was found as 1.08 (*VIF*> 5; *benchmark*), so, there is no any possibility of multicollinearity in the data (Mansfield and Helms, 1982; Kalnins, 2018).

**Table 3:** Correlation and multicollinearity test.

	**1**	**2**	**3**	**4**	**Collinearity statistics**	
					**Tolerance**	**VIF**
OI (OI)	1				0.926	1.080
Perceived inside status (PIS)	0.133^*^	1			0.977	1.023
IWB (IWB)	0.272^**^	0.271^**^	1			
PP(PP)	0.251^**^	0.105	0.244^**^	1	0.932	1.073

*Collinearity statistics; variance inflation factor (VIF)*.

*Generated through SPSS*.

* *p < 0.005,*

** *p < 0.01*.

### Development of Structural Equation Modeling (SEM)

#### Exploratory Factor Analysis Check

Anderson and Gerbing (1988) presented a two-step approach; the first step was exploratory factor analysis (EFA), and the second was confirmatory factor analysis (CFA). The study ran all the assumptions of EFA to get an adequate level of parametric data and item validity for measurement and a structural model. The assumptions started from Kaiser-Meyer-Olkin (*KMO* = 0.93), and the threshold is (0.8 > *KMO* < 1), *which* presented a marvelous value of sample adequacy, so the partial correlation between the variables was strengthened (Kaiser, 1974). The correlation matrix of the study data is not identical because the null hypothesis of Bartlett's Test of Sphericity is rejected, which establishes that the study variables proved correlated. The data have been rotated for orthogonal (varimax) rotation to extract the fundamental factors (Hayton et al., 2004). The criteria of (eigenvalues = 1) were adopted to verify the count of extracted factors; based on the criteria, four factors achieved >1. The rotation analysis provided exactly four variables. Table 4 indicates that four study factors were found to explain the 69.281% variation, which is considered a good attempt. Items loadings also achieved the acceptable level of thresholds and fell between (*Item loadings*:0.729 *to* 0.882), which exceeded the criteria value of 0.6 (Scheaf et al., 2020).

**Table 4:** Rotated component matrix.

**Construct**	**Kaiser-Meyer-Olkin measure of sampling adequacy**				**0.925**		
	**Bartlett's test of sphericity**				**0.000**		
	**Initial eigenvalues**	**10.165**	**5.988**	**4.567**	**2.827**		
	**Total variance explained**	**20.224**	**40.216**	**59.597**	**69.281**		
	**Items**	**1**	**2**	**3**	**4**	**Mean**	**Std. deviation**
Proactive personality	PP1	0.804				3.830	0.8915
	PP2	0.771				3.827	0.8595
	PP3	0.729				3.677	0.8831
	PP4	0.814				3.677	0.9025
	PP5	0.843				3.801	0.9080
OI	OI1		0.844			3.827	0.9306
	OI2		0.832			3.902	0.9102
	OI3		0.776			3.841	0.9222
	OI4		0.778			3.856	0.9136
	OI5		0.821			3.844	0.9367
	OI6		0.827			3.867	0.9026
	OI7		0.840			3.790	0.9244
	OI8		0.818			3.879	0.9074
	OI9		0.836			3.899	0.8955
	OI10		0.770			3.942	0.9105
PIS	PIS1			0.845		4.156	0.8287
	PIS2			0.822		4.150	0.8227
	PIS3			0.821		4.084	0.8544
	PIS4			0.809		4.058	0.8097
	PIS5			0.809		4.118	0.8537
	PIS6			0.831		4.084	0.7689
	PIS7			0.799		4.020	0.8977
	PIS8			0.822		4.029	0.8183
	PIS9			0.767		4.000	0.9028
	PIS10			0.804		4.101	0.8037
IWB	IWB1				0.793	3.597	0.8661
	IWB2				0.810	3.654	0.9068
	IWB3				0.849	3.617	0.9187
	IWB4				0.828	3.620	0.9614
	IWB5				0.845	3.597	0.9242
	IWB6				0.834	3.582	0.9349
	IWB7				0.830	3.605	0.9449
	IWB8				0.882	3.640	0.9465
	IWB9				0.852	3.631	0.9167

*EFA through principal component method analysis (PCA)*.

*Generated through SPSS*.

#### Confirmatory Factor Analysis Check

CFA is the second step of that approach mentioned above in EFA. To test the model fitness concerning data, CFA has been conducted. Table 5 results revealed an acceptable fit; the study's data achieved an excellent model fitness after minor revision. All the absolute and relative measures have achieved the threshold level of significance (χ2, CMIN/DF = 1 > 1.953 < 3, CFI = 0.953 > 0.95, SRMR = 0.04 < 0.08 and RMSEA = 0.05 < 0.06) (Hu and Bentler, 1999).

**Table 5:** CFA model fit indices.

		**CFA model fit**		**Revised CFA model fit**	
**Measure**	**Acceptable values**	**Estimate**	**Interpretation**	**Estimate**	**Interpretation**
CMIN/DF	Between 1 and 3	2.337	Excellent	1.924	Excellent
CFI	>0.95	0.926	Acceptable	0.953	Excellent
SRMR	<0.08	0.042	Excellent	0.04	Excellent
RMSEA	<0.06	0.062	Acceptable	0.05	Excellent

*CMIN, chi square fit; DF, degree of freedom; CFI, comparative fit index; RMSEA, root mean square error of approximation; SRMR, standardized root mean square residual*.

*Generated through AMOS*.

#### Reliability and Validity Check

Cronbach's alpha and CFA were utilized to determine the instrument reliability and validity where all the alpha values ranged from 0.867 to 0.954 above 0.70 (Cronbach, 1951), while the values of composite reliability (CR) ranged from 0.868 to 0.954 beyond the threshold of 0.6 (Kahle and Malhotra, 1994). Consequently, the results of alpha and CR provided strong evidence of internal consistency.

The study has analyzed three types of validity, and the findings were displayed in Table 6: firstly, all the factor's loadings have exceeded the cutoff value of 0.50, achieving the convergent validity of data (Martin and Rubin, 1995; Rosenbach et al., 2009). Secondly, the values of average variance extracted (AVE) (0.570–0.641) were above the threshold of 0.50 and below the values of MSV, which supported the divergent/discriminant validity (Fornell and Larcker, 1981; Ab Hamid et al., 2017). Thirdly, the convergent validity verified the correlation within the factors, and discriminant validity assured the correlation with similar measures of the concept from the existing literature; hence, nomological validity has been observed. Finally, nomological validity played a pivotal role in the model's robustness and associated the theoretical model with the structural model (Spiro and Weitz, 1990; Haynie and Shepherd, 2009).

**Table 6:** Validityand reliability.

**Construct**	**Items**	**CR**	**Alpha**	**AVE**	**MSV**	**MaxR**	**1**	**2**	**3**	**4**
OI	10	0.948	0.867	0.648	0.079	0.950	0.805			
PIS	10	0.947	0.948	0.641	0.080	0.948	0.141^*^	0.800		
IWB	09	0.954	0.946	0.700	0.080	0.956	0.280^***^	0.283^***^	0.836	
PP	06	0.868	0.954	0.570	0.074	0.873	0.273^***^	0.103	0.259^***^	0.755

*CR, Composite reliability; alpha, Cronbach's alpha; AVE, average variance extracted*.

*Generated through AMOS*.

* *p < 0.005,*

*** *p < 0.001*.

### Results and Findings

#### Direct and Indirect Relations Testing

Table 7 shows that the SEM model established a good fit of the study data, (χ2, CMIN/DF = 1 > 1.85 < 3, CFI = 0.948 > 0.95, SRMR = 0.057 < 0.08 and RMSEA = 0.05 < 0.06) (Hu and Bentler, 1999). It has provided the confidence to analyze the structural model, including all the variables.

**Table 7:** SEM model fit indices.

		**SEM Model Fit**	
**Measure**	**Acceptable values**	**Estimate**	**Interpretation**
CMIN/DF	Between 1 and 3	1.850	Excellent
CFI	>0.95	0.948	Acceptable
SRMR	<0.08	0.057	Excellent
RMSEA	<0.06	0.050	Excellent

*CMIN, chi square fit; DF, degree of freedom; CFI, comparative fit index; RMSEA, root mean square error of approximation; SRMR, standardized root mean square residual*.

*Generated through AMOS*.

**Figure 3:**
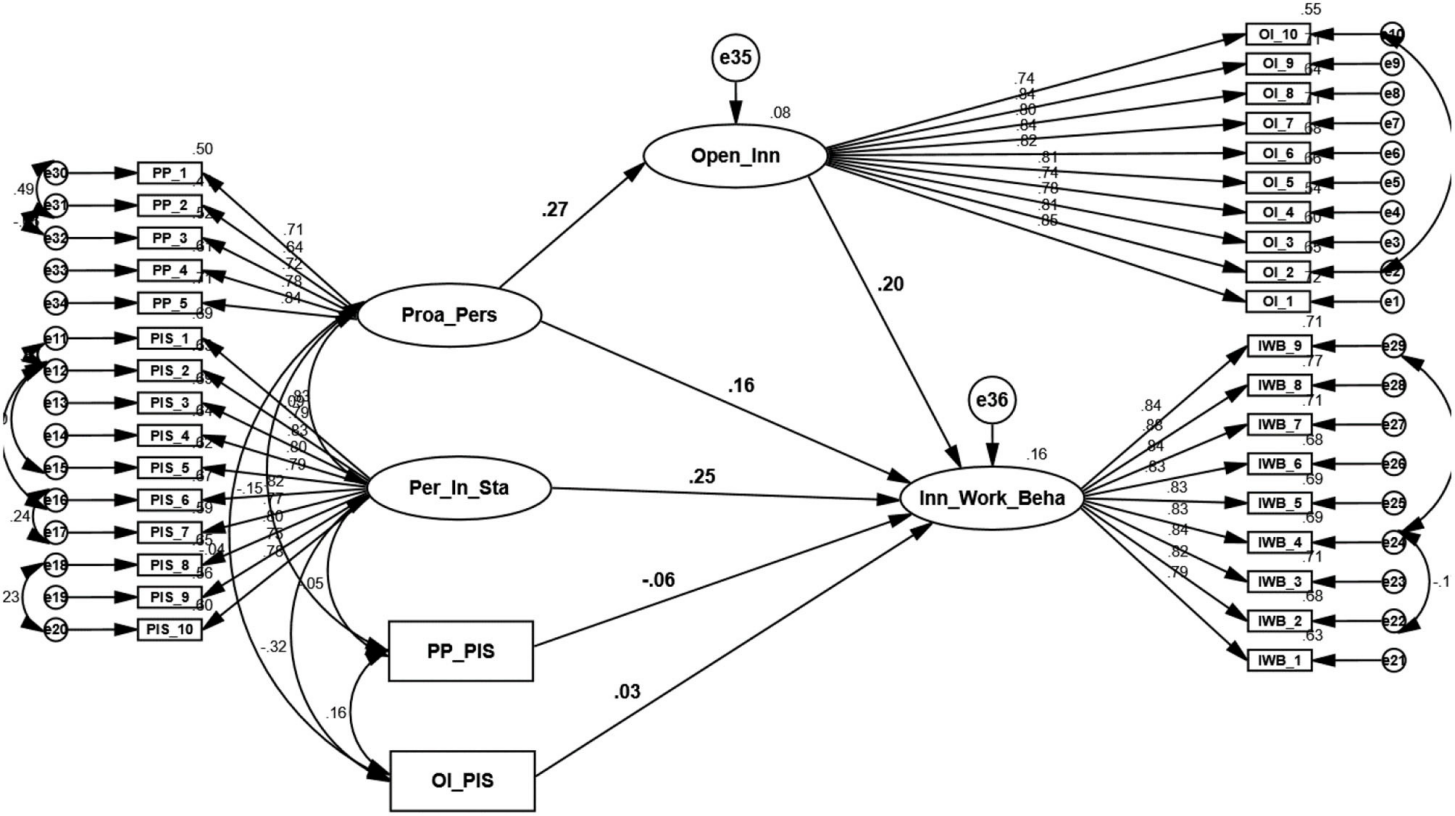
SEM Model. Source: generated through AMOS.

The H5 and H6 have targeted hypotheses of the study to investigate direct and indirect mediated moderation, respectively. That coherent model needs to establish some assumptions tested in H1, H2, H3, and H4. In contemporary research, Hayes presented the most appropriate technique to test mediated moderation through bootstrapping technique (Hayes, 2013). Based on this technique, to examine the targeted hypotheses, we have developed and used a user-defined estimate and ran this coherent model in one shot through AMOS, and the results are presented in Table 8 and Figure 3.

**Table 8:** Hypothesis testing.

							**Bootstrapping percentile**				
**Hypotheses**	**Label**	**Path**	***R*** ^**2**^	**β**	**T**	**ρ**	**β**	**LL**	**UL**	**ρ**	**Decision**
H1	C1	PP→ IWB	0.163	0.17	2.75	0.020	0.16	0.024	0.306	0.020	Accepted
H2	A	PP→ OI	0.274	0.34	4.54	^***^	0.27	0.139	0.403	0.001	Accepted
H3	B1	OI→ IWB	0.203	0.17	3.63	^***^	0.20	0.058	0.329	0.006	Accepted
H4	Mediation	PP→ OI→ IWB		0.06		0.006					Partial

*Label; paths identified in syntax, Bootstrapping; 5,000 and 5% CI*.

*LL, lower limit; UL, upper limit*.

Bootstrap sample size = 5,000, ^*^ p < 0.005, ^**^ p < 0.01,

*** *p < 0.001*.

*Self-generated*.

The H1 propelled positive relationship between PP and IWB presented by Label C1. Results (β = 0.17, *T* = 2.75, ρ = 0.02) demonstrated that IWB was significantly influenced by PP. One unit increase in PP brought a 0.17 unit increase in IWB. The value of (*R*^2^ = 0.163) provided 16.3% explained variation in IWB through PP. So, the study has accepted the H1 based on the above results. The H2 proposed positive relatedness between PP and OI presented by Label A. Results (β = 0.34, *T* = 4.54, ρ = ***) reported that the outcome variable OI was significantly impacted by the predictor PP, although an increase of one unit in a predictor led 0.34 unit increase in the outcome variable. Regression value of (*R*^2^ = 0.274) explained 27.4% variation among outcome variables of OI. Hence, the H2 has been accepted after analysis.

The H3-motivated, positive relation of OI with IWB is depicted by Label B1. Quantitative analysis (β = 0.17, *T* = 3.63, ρ = ***) tested that IWB was significantly influenced by the OI. Therefore, one unit increase in OI transferred 0.17 unit's enhancement in IWB. The *R*^2^ achieved value of (*R*^2^ = 0.203), which explained the 20.3% variation of the dependent variable among IWB. So, a study has accepted H3 based on the results. The H4 proposed a mediation effect of OI between PP and IWB. The mediation analysis has been tested through 5,000 percentile-bootstrapping and 95% internal confidence (Preacher and Hayes, 2008). H1 has tested the direct effect and resulted in a positively significant relationship. Then paths (a) and (b), H2 and H3, respectively, proved positively significant. However, the change in indirect impact was less than direct impact, but it was different from zero. Hence, partial mediation has been observed based on the Hayes mediation technique. Consequently, it has been established that the relationship between PP and IWB has been partially mediated by the OI and increased the impact of the predictor on the outcome variable through mediation.

#### Conditional Indirect and Direct Effects Testing

**Figure 4:**
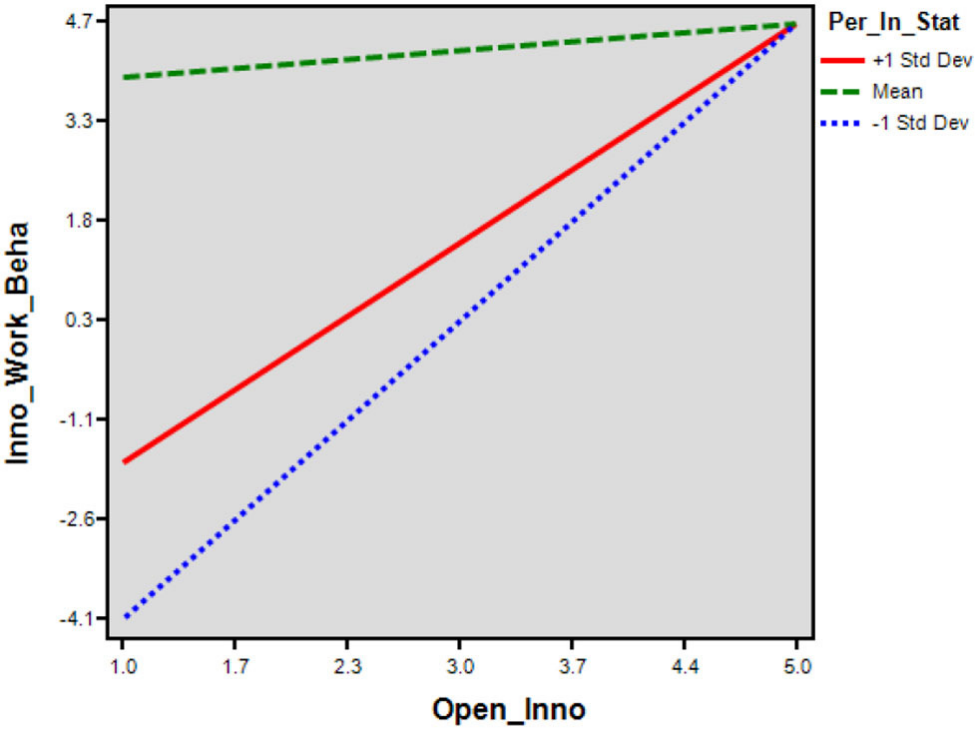
Indirect effects. Source: generated through interaction software.

**Figure 5:**
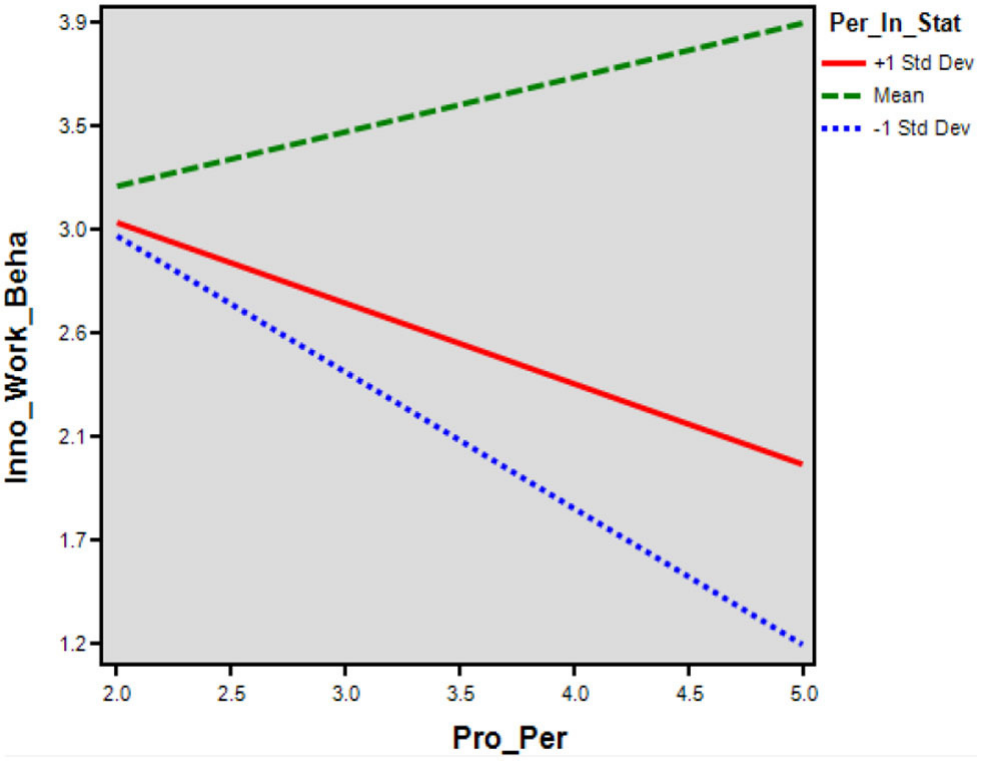
Direct effects. Source: generated through interaction software.

The indirect interactional effect of OI and PIS (β = 0.18, *T* = 2.96, ρ = 0.02) on endogenous IWB was significantly positive and reported a mediated moderation. The value of (*R*^2^ = 0.091), for conditional indirect effect explained a 9.1% variation among endogenous. The moderation effect of PIS on OI and IWB was demonstrated in Figure 4. The moderating relationship between OI and IWB resulted (β = 0.064, ρ = 0.001) significantly stronger for the employees having a higher level of PIS (+ 1SD) opposite to a lower level (−1SD) (β = 0.056, ρ = 0.005 ) (Table 9). The moderation power analysis was reported (*F*^2^ = 0.66, λ = 230.27, *Power* = 0.076), where *F*^2^ presented the effect size, λ demonstrated the non-centrality parameter, and power presented the model-observed power. The more value of λ leads to increased observed power; hence, the moderation effect is significant and powerful (Table 10). While the direct interactional effect of PP and PIS (β = −0.06, *T* = −1.18, ρ = 0.375) on endogenous IWB was negatively insignificant and did not report a mediated moderation, the moderation effect of PIS on PP and IWB was demonstrated in Figure 5. The moderating relationship between PP and IWB resulted (β = 0.154, ρ = 0.072) insignificant for the employees having a higher level of PIS (+ 1SD). The moderation power analysis reported (*F*^2^ = 0.16, λ = 55.96, *Power* = 0.00). The more value of λ leads to a decrease in the observed power.

**Table 9:** Conditional direct and indirect effects.

	**User-defined estimands**								
**Parameter**	**Bias-corrected bootstrapping 95% CI**					**Percentile bootstrapping 95% CI**			
	**SE**	**SE-SE**	**Mean**	**Bias**	**SE-Bias**	**β**	**Lower**	**Upper**	**ρ**
Conditional indirect effect of OI with PIS at low, medium and high mean:									
IndEffLow1	0.028	0.0.00	0.053	0.004	0.001	0.056	0.014	0.107	0.005
IndEffMedium1	0.024	0.000	0.058	0.002	0.000	0.060	0.016	0.108	0.006
IndEffHigh1	0.026	0.000	0.063	0.001	0.000	0.064	0.018	0.119	0.001
Conditional direct effect of PP with PIS at low, medium and high mean:									
DireEffLow1	0.083	0.001	0.205	0.002	0.002	0.204	0.045	0.373	0.016
DireEffMedium1	0.075	0.001	0.176	−0.001	0.001	0.177	0.028	0.324	0.020
DireEffHigh1	0.081	0.001	0.147	−0.003	0.001	0.150	−0.012	0.309	0.072
**Mediated Moderation**									
**Hypothesis**	**Label**	**Path**	***R*** ^**2**^	**T**	**β**	**Lower**	**Upper**	**ρ**
H5	B2	OI_X_PIS→ IWB	0.091	2.96	0.18	0.031	0.320	0.022
H6	C3	PP_X_PIS→ IWB	−0.062	−1.18	−0.06	−0.221	0.077	0.375

*SE, standard error*.

*Bootstrapping: 5,000 and 95% CI*.

*LL, lower limit; UL, upper limit; CI, confidence of interval*.

*Self-generated*.

**Table 10:** Moderation model power analysis.

	**Effect size** ***F***^**2**^	**Noncentrality parameter** **λ**	**Critical F**	**Noncentral F**	**Beta (type II error rate)**	**Observed power**
Conditional indirect effect:	0.66	230.27	12.8	7.86	0.25	0.76
Conditional direct effect:	0.16	55.96	15.94	0.69	0.00	0.00

*λ: Noncentrality parameter*.

*Generated through interaction software*.

Hayes (2015) and Khan et al. (2021) presented a comprehensive technique based on categorical assumptions to test mediated moderation. They have elaborated on these assumptions; (a) the direct relationship between exogenous and endogenous should be significant; (b) the relationship between the mediator and endogenous should be significant; (c) the interactional effect between the mediator and moderation on endogenous should be significant; (d) the strength of the conditional indirect effect should be different at high, medium, and low levels; (e) the interactional effect between exogenous and a moderator on endogenous should be significant and, finally, (f) the strength of the conditional direct effect should be different at high, medium, and low levels.

The empirical analysis evidenced that H1 (β = 0.17, *T* = 2.75, ρ = 0.02) has met assumption (a). The H3 (β = 0.17, *T* = 3.63, ρ = ***) has reached the (b) assumption of the suggested technique. The H5 has been accepted in the above analysis, which has fulfilled the (c) assumption and attained the different levels of strength and satisfied the (d) assumption. Hence, the conditional indirect effect of OI and PIS on IWB has been proved positively significant, which led to the acceptance of H5. We can conclude based on the results that the strength of moderated mediation of OI and perceived-insider status has achieved a higher level of perceived-insider status in IWB of the employees in the IT sector of China. We concluded based on the results that PIS moderated the strength of the indirect relationship between PP and IWB through the mediation of OI. The relationship proved significant for the employees having a higher level of PIS than those who are lower PIS in the IT sector of China.

To test the H6, results of indirect effect were negatively insignificant, which has failed to achieve the (e) assumption. However, the results fulfilled the (f) assumption but proved the direct effect's negative and weaker level of strength. Hence, the moderation effect proved insignificant and weaker, so H6 has been rejected. The study suggested that PIS did not moderate the strength of the direct relationship between PP and IWB through the mediation of OI in the IT industry of China.

## Discussion

### Theoretical Implications

This study contributes significantly to the existing literature on the IT sector in multi-dimensions. Firstly, this study stretches the broaden-and-build theory to the IT sector with the juxtaposition of innovation theory. To empirically test such a blended theoretical model in the literature of management and organizational behavior studies happened for the first time, we found no evidence in the literature related to the assimilation of the theory of innovation and the broaden-and-build theory. Many studies happened in the IT sector emphasized technological and process innovation. Unfortunately, these studies concealed or marginalized the role of behavior in innovative work. So, studying broaden-and-build theory parallel to the innovation theory has filled this covert gap (Fredrickson, 1998).

Truxillo et al. (2012), Alikaj et al. (2021), Ruiz Moreno et al. (2021), and Zhang et al. (2021) antecedents have idealized the positive impact of PP on employee outcomes but did not unfold its impact on IWB. Our study contributes to this overt literature gap by adding empirical evidence that PP has a significant positive impact on IWB in the IT sector of China. Indeed, the deliberate interventions for OI supported the cultural developments to support OI in the workplace. As a result, the OI allows the sharing of ideas at internal and external levels and ultimately leads to IWB. This study establishes a positive relationship between PP and OI, and OI and IWB.

Secondly, Liu et al. (2017), Ahmad et al. (2021a), Pan et al. (2021), and Hoang et al. (2022) studied trust in leadership, psychological safety, affective states, creative self-efficacy, error management climate, and self-efficacy as mediators between PP and IWB. The current study investigates partial mediation of OI between the relationship between PP and IWB. OI was studied many times in the context of leadership roles, and few studies elaborated on its multiple perspectives (Edelbroek et al., 2019). Our study unfolds the significance of OI in the context of innovation theory and starts a debate on the new perspective of OI.

Thirdly, Dionne et al. (2002); Caron et al. (2019); and Kang et al. (2021) antiquities have discussed the different roles of PIS as a significant outcome variable as a mediator and found no mediation, somewhere found a partial mediation and as a predictor with IWB. Indeed, there was a question mark on the well-defined role of PIS, so our study attempts to investigate PIS's role as a moderator with conditional direct and indirect effects on IWB. PIS contributes to the scholarly debate and establishes a significant conditional effect with the mediator. Hence, this study initiates the new journey of PIS in the literature on management and organizational behavior from the perspective of the IT sector.

Finally, this study unfolds the explicit relationships between this coherent model to enhance the literature through the mediated moderation of OI and PIS between PP and IWB to explore the new horizons about the future of innovative products and services in the IT sector. Although the indirect effect of mediated moderation tested a significantly higher strength of PIS on IWB, however, the direct effect results insignificantly. Furthermore, this study illuminates the dark path of IWB through the spread of OI and PIS. This study highlights that PP plays a dual role through the optics of theories and defines the IWB more strongly, which has not been examined in the previous literature.

### Managerial Implications

Our study puts some exceptional implications for managers to address the problems related to management and organizational settings in the IT sector. Our results propel that IT-related organizations concerned with developing IWB of their employees would earn marvels by placing employees with proactive personalities. Simultaneously, they must develop a culture that OI must support to enhance the knowledge and skill levels of the employees through the sharing of ideas. IT-based organizations could get more innovative products and services if they considered the significance of a higher level of employees' PIS.

The study has indicated that the interplay of OI and PIS indirectly influences employees' IWB, enabling them to produce creativity in their work, although managers have a pivotal role to play here through the forceful implications of intervening variables on employees' emotions that would lead to increased IWB according to the broaden-and-build theory.

The managers of IT sector organizations should redefine their recruitment policy and consider selecting employees with higher levels of proactive personalities to align their skill levels with OI. This stuff of employees would be more comfortable with the innovation knowledge sharing and bring innovation in their work according to the optic of the theory of innovation. The creation of innovation is the primary goal of IT-based organizations to sustain the market share and achieve a competitive advantage. Managers must consider the behavioral dimension of the employees with the traditional techniques based on competition in the IT sector. However, this study exposed that employees' ability to produce innovation is the direct function of a higher level of PIS.

### Conclusion

The conclusion of our research elucidates the connections and interplay of the variables *via* employees' proactive personalities that can affect their IWB. This study deals with the complex phenomenon of innovation in the IT sector through technological and behavioral innovation tools with the theory of innovation and broaden-and-build theory, respectively. However, many studies have investigated the IT sector for the impactful application of innovation from functional or behavioral perspectives. This study confronts the issue with the robust model of mediated moderation between the relationship between employees' proactive personalities and their IWB. The combined influx of the purported theories to address management studies phenomenon in the IT sector permeates the covert stream for debate.

### Limitations and Future Research

It is widely believed that nothing is perfect in this world, so our study is not without gaps and limits. It has some traditional and emerging limitations. Firstly, according to the traditional perspective, we used to collect data cross-sectional; indeed, in the future, the temporal data could present a higher level of the precision of the results. Furthermore, the research universe was limited to China only, affecting our results' generalizability. The application of this study could enhance results precision after being tested in diversified cultural and organizational settings. Testing this coherent model in the hospitality sector could unfold exciting facts in the existing literature.

Secondly, according to the emerging perspective, the study respondents were individuals. Therefore, the data could be collected from groups to associate the PP of individuals with groups. Finally, extending the theoretical scope of the study from IWB to sustainable organizational development could open a new discussion in organizational development literature. Our study introduced a theoretical framework to test and develop the theory through a general regression model; future studies can use the partial least square method to predict this theoretical framework.

## Data Availability Statement

The raw data supporting the conclusions of this article will be made available by the authors, without undue reservation.

## Ethics Statement

The studies involving human participants were reviewed and approved by Weifang University, Weifang, Shandong, China. The patients/participants provided their written informed consent to participate in this study.

## Author Contributions

WL and SG proposed the research idea, analyzed the results, and wrote the manuscript. YW, MAS, and MRS designed and carried out the methodology and extensively edited the manuscript. All authors contributed to the article and approved the submitted version.

## Conflict of Interest

The authors declare that the research was conducted in the absence of any commercial or financial relationships that could be construed as a potential conflict of interest.

## Publisher's Note

All claims expressed in this article are solely those of the authors and do not necessarily represent those of their affiliated organizations, or those of the publisher, the editors and the reviewers. Any product that may be evaluated in this article, or claim that may be made by its manufacturer, is not guaranteed or endorsed by the publisher.
